# Probing the intrinsic mechanism and evolution characteristics of online shopping customer satisfaction via text mining of online reviews

**DOI:** 10.1371/journal.pone.0321202

**Published:** 2025-05-07

**Authors:** Mingyue Wang, Rui Kong, Yibo Wang

**Affiliations:** 1 School of Economics and Management, China University of Geosciences, Beijing, China; 2 Key Laboratory on Resources and Environment Capacity under Ministry of Land and Resources of People’s Republic of China, Beijing, China; University of Naples Federico II: Universita degli Studi di Napoli Federico II, ITALY

## Abstract

Prior research has tended to disregard the dynamic nature of customer satisfaction in online shopping and how it influences corporate marketing decisions. This study originally introduces a dynamic online shopping customer satisfaction index model and devises a new text mining algorithm to quantify online reviews, testing and analyzing the model to reveal the intrinsic mechanism and evolutionary characteristics of online shopping customer satisfaction. Findings reveal disparities between the online shopping customer satisfaction index model and the American customer satisfaction index model. Specifically, customer expectations significantly impact customer loyalty, while customer loyalty influences complaint rates. The study also highlights the impact of COVID-19, which has intensified competition and underscored the importance of perceived quality and brand image. Our findings provides a reference for e-commerce enterprises to realize data-driven marketing decisions.

## 1. Introduction

The size of the e-commerce market has increased as a result of the advancement of Internet technology [1]. China’s online retail sales reached 15.4 trillion yuan in 2023, up 11% from the previous year [2]. The COVID-19 pandemic has raised the rate of e-commerce penetration and intensified competition in the e-commerce sector [3]. To address these challenges, e-commerce businesses must accurately satisfy customer needs, boost customer satisfaction, and take up a larger portion of the market. Among these challenges, the issue of how to increase customer satisfaction has emerged as the most crucial.

China is undergoing rapid changes and facing emerging demands, which compels enterprises to identify more effective and advanced approaches to enhance customer satisfaction. Due to societal development, increasingly diverse customer demands, and ongoing technical progress, customer satisfaction management has entered a phase of delicacy management. This poses new challenges for e-commerce companies in accurately improving customer satisfaction [4]. Additionally, variations in the composition of online shopping customers and shifts in the business environment lead to evolving factors and their varying impact on customer satisfaction. As a result, investigating the evolving characteristics of the intrinsic mechanisms of online shopping customer satisfaction is critical for achieving refined customer satisfaction management.

Experts from Sweden, the United States, and Europe have put forward the theory of customer satisfaction earlier and popularized it worldwide [5]. Many scholars have studied the intrinsic mechanism of online shopping customer satisfaction and identified the factors affecting the improvement of customer satisfaction based on it [6,7]. However, the intrinsic mechanism of online customer satisfaction changes over time [8]. This will affect the enterprise’s future marketing strategy and financial performance [9,10].

From prior research, two key gaps have emerged. Firstly, the dynamic nature of intrinsic mechanism shaping online shopping customer satisfaction has been overlooked, despite their pivotal role in devising marketing strategies for e-commerce enterprises. Secondly, while previous studies acknowledged the significance of online reviews in influencing online shopping customer satisfaction, they rarely harnessed text mining techniques grounded in these reviews to unveil such intrinsic mechanism. However, leveraging online reviews facilitates a more comprehensive, genuine, and timely capture of customer feedback.

To bridge the research gap, this study proposes a dynamic Online Shopping Customer Satisfaction Index (OSCSI) model. Distinct from current models, it introduces time as a moderating variable to capture the evolution characteristics of customer satisfaction. By incorporating time dynamics, OSCSI offers a more nuanced understanding of how customer satisfaction evolves and how to manage it in the rapidly changing e-commerce environment effectively. Additionally, a novel text mining algorithm is developed to convert textual data into scale data in real-time, offering researchers a valuable tool for analysis. Differing from previous research methodologies, this paper adopts an innovative approach that combines management models with text mining technology.

The rest of this research is organized as follows: The theoretical background and hypothesis are covered in Section 2. Methods and data are described in Section 3. Section 4 exhibits the results. The discussion and implications are further developed in Section 5, respectively. Section 6 presents the conclusions.

## 2. Literature review and theoretical foundation

### 2.1. Customer satisfaction index model

The Swedish Customer Satisfaction Barometer (SCSB) is the first customer satisfaction index model [11]. Fornell et al. (1996) introduced the American Customer Satisfaction Index (ACSI), which incorporated the element of perceived quality into the SCSB [12]. Subsequently, the European Customer Satisfaction Index (ECSI) was established in 2000 by the European Union, drawing on the SCSB and ACSI while considering the European context [13]. The ECSI removed the customer complaints element and introduced the corporate image element. The Chinese Customer Satisfaction Index (CCSI) was jointly proposed by the China Institute of Standardization and Tsinghua University in 2002. The CCSI removed customer complaints and expectations while introducing expected quality and brand image elements. These developments significantly enriched the understanding of customer satisfaction by adapting the index models to different cultural contexts and incorporating key elements that reflect customer perceptions.

**Fig 1:**
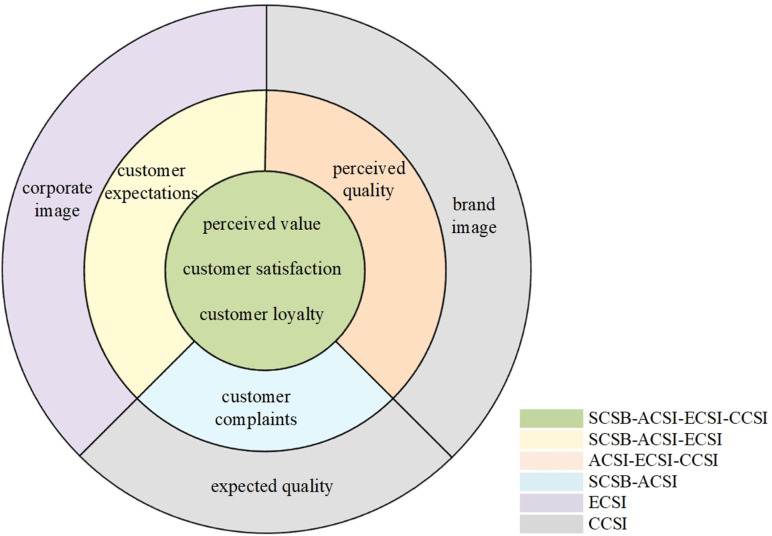
Comparison of the elements of the Customer Satisfaction Index (CSI) model.

Fig 1 illustrates the composition of the four models based on included elements. The innermost layer consists of three elements present in all four models. The middle layer includes elements shared by at least two models, while the outermost layer comprises elements exclusive to a single model.

Online shopping differs from traditional shopping in terms of delivery methods, trading platforms, and payment methods [14–16]. The drivers of satisfaction between online and in-store customers are different [17]. Studies on the intrinsic mechanism of online shopping customer satisfaction are mostly based on ACSI or ECSI [18,19]. Previous studies have examined platforms such as PChome Online, Meituan and Tmall [19–21].

Online shopping customer satisfaction is influenced by various factors beyond those included in the 4 established customer satisfaction models. For example, the online shopping platform image affects customer trust and satisfaction, as do product brand images [22–24]. Both are exogenous factors that impact customer satisfaction [25]. To ensure a comprehensive assessment of online customer satisfaction, we have integrated all elements from the established models into OSCSI model.

Online reviews, as a manifestation of electronic word of mouth, have substantial mining value in exploring customer satisfaction. Social commerce constructs play a pivotal role in influencing customer trust and bolstering purchase intentions [26]. Buyers’ assessments of products or services, along with the online shopping platform’s reputation, significantly influence how customers behave when shopping online [27]. Electronic word of mouth affects online shopping customer satisfaction and loyalty [28]. Customer satisfaction also affects word-of-mouth communication and consumer behavior [29]. Leveraging online reviews and text mining, researchers have pinpointed factors shaping customer satisfaction in online shopping [30,31].

Time is a crucial factor that impacts customer satisfaction. The approach to improving customer satisfaction is not set in stone, as customer satisfaction is dynamic [32]. In terms of research content, this includes analyzing the dynamic changes in customer satisfaction to develop future product improvement strategies and constructing a real-time monitoring system to provide early warnings aimed at enhancing customer satisfaction [33,34].

In summary, research on customer satisfaction index models is well-developed. Many scholars have expanded existing customer satisfaction index models by incorporating various elements based on the characteristics of the research subjects. Moreover, some scholars have acknowledged the dynamic nature of customer satisfaction and incorporated time factors into the analysis of customer satisfaction. Based on these insights, the paper will develop a dynamic OSCSI model to more comprehensively reflect changes in customer satisfaction over time. Subsequently, a detailed analysis of hypothesis development will be provided.

### 2.2. Hypotheses development

Many studies have explored the intrinsic mechanisms of customer satisfaction with ACSI and ESCI being widely used. ACSI is considered more authoritative due to its high representativeness [35]. However, its reliance on static, structured questionnaire measurements makes it less suitable for dynamic online shopping satisfaction models based on online reviews. In addition, online reviews capture key elements of CSI, such as perceived quality, perceived value, customer expectations, loyalty and so on [36]. Based on these elements, we propose hypotheses that better reflect the dynamic nature of OSCSI.

Perceived quality refers to consumers’ subjective perception and evaluation of the quality of purchased products or services. According to the Expectation Confirmation Theory (ECT) and the Latent Dirichlet allocation (LDA) theme modeling results, perceived quality is considered as a core factor affecting customer satisfaction [37,38]. In addition, perceived quality, as a key component of perceived value, directly affects the customer’s perception of the overall value of the product [39]. Based on these insights, we propose the following hypothesis:

H1a: Perceived quality directly affects customer satisfaction.

H1b: Perceived quality directly affects perceived value.

Definitions of perceived value have been quite prolific. This paper adopts the classic definition of perceived value, which refers to the perception of value in terms of costs and benefits. Purchasing products through e-commerce platforms offers good cost performance, which enhances perceived value [40]. Compared to offline stores, products on e-commerce platforms are typically more cost-effective, which attracts more customers to shop online. Prior studies have confirmed the direct impact of perceived value on customer satisfaction at both theoretical and empirical levels [41]. However, in online shopping for electronic products, the buyer’s decision-making process is often complex. As a result, perceived value may have a limited effect on customer satisfaction. This observation is supported by research in the context of e-grocery shopping [42].

Perceived value is classified into utilitarian and hedonic dimensions. The perceived value defined in this study is embedded in the utilitarian dimension. It is demonstrated that millennials place greater emphasis on hedonic value than on utilitarian value [43]. Furthermore, another study indicates that hedonic value has a more significant impact on customer satisfaction, while functional value has a greater influence on customer loyalty [44]. According to the Just-World Hypothesis, when customers perceive a higher value in online shopping, they tend to take it for granted. This increases their willingness to engage in online shopping and trust the process [45]. As a result, customers are more likely to repurchase and recommend the online store to others [46]. An elevated perceived value boosts customer loyalty by increasing their tendency to repurchase [47]. Therefore, we propose hypothesis 2.

H2: Perceived value directly affects customer loyalty.

As outlined in the ECT and perceived value theory, customer expectations directly affect both perceived quality and perceived value, as reflected in the ACSI. Furthermore, the ACSI framework indicates that customer expectations also affect customer loyalty through satisfaction. However, some customers may form loyalty based on long-term expectations of a brand, which can occur independently of satisfaction. For instance, customers of an e-commerce platform expect it to provide prompt delivery and convenient services. If the platform consistently meets these expectations, customers will develop long-term loyalty that may not always be rooted in satisfaction with individual purchase experiences, as suggested by brand commitment theory. In difficult-to-evaluate services, service value expectations ultimately determine loyalty [48]. The direct effect of customer expectations on customer loyalty has been validated in the hospitality industry and boarding school settings [49,50]. Thus, hypothesis 3 is proposed.

H3a: Customer expectations directly affect perceived quality.

H3b: Customer expectations directly affect perceived value.

H3c: Customer expectations directly affect customer satisfaction.

H3d: Customer expectations directly affect customer loyalty.

According to the marketing literature, customer satisfaction is recognized as a key factor in evaluating the quality of the relationship between service providers and users [51]. Satisfied customers tend to have fewer complaints [52]. However, the relationship between customer satisfaction and complaints is more complex in online shopping contexts, particularly in review-based studies. Even when customers are satisfied, they may still express dissatisfaction in their reviews, especially when their expectations are not met [53]. Moreover, customer satisfaction is considered a core element of customer retention. It reflects how well a company’s product meets or exceeds customer expectations, enhances the relationship between customers and service providers, and drives online repurchase behavior [54]. Hypothesis 4 is formulated accordingly.

H4a: Customer satisfaction directly affects customer complaints.

H4b: Customer satisfaction directly affects customer loyalty.

ACSI suggests that customer complaints have a direct negative impact on customer loyalty. Loyal customers usually have higher expectations, making them more likely to complain when issues arise, thereby motivating companies to improve. In addition, individuals tend to express their negative emotions during close social interactions [55]. This tendency is further illustrated by a study showing that Chinese customers are more likely to express dissatisfaction to companies with which they have established a positive relationship [56]. Drawing on insights from a collectivist cultural context, loyal customers are more likely to complain when dissatisfied than non-loyal customers [57]. Hence, hypothesis 5 is formulated.

H5: Customer loyalty directly affects customer complaints.

In addition to the factors mentioned above, the ECSI and CCSI introduce two exogenous variables—brand image and corporate image. In the context of online shopping, these correspond to product brand image and online platform image, respectively. Product brand image refers to the overall perception of the brand in the customer’s mind and plays a direct role in perceived quality. A positive product brand image has been found to be associated with higher levels of perceived quality [58,59]. Through publicity, advertising and brand story, a brand conveys a series of promises that shape the brand image, directly affecting customer expectations [60]. In other words, companies shape customer expectations through product brand image. Therefore, the following hypotheses are proposed.

H6a: Product brand image directly affects perceived quality.

H6b: Product brand image directly affects customer expectations.

Online shopping platforms tend to have different products and market niches as a shopping medium. Therefore, this study proposes that the online shopping platform image, akin to corporate image, exerts an indirect influence on customer satisfaction by shaping perceived quality [13]. The reputation of online shopping platforms influences customer loyalty [61]. Additionally, the online shopping platform image has a direct and significant positive impact on customer expectations and customer loyalty [62,63]. Accordingly, the following hypotheses are put forward.

H7a: Online shopping platform image directly affects perceived quality.

H7b: Online shopping platform image directly affects customer expectations.

H7c: Online shopping platform image directly affects customer loyalty.

The National Center for Quality Research at the University of Michigan Business School and the American Society for Quality have conducted yearly studies of customer satisfaction indices by industry since 1994. Some scholars have analyzed the impact of customer experience levels on customer satisfaction and explored how these factors change over time [8,64]. Other researchers have developed a dynamic evaluation and measurement system for online shopping customer satisfaction based on online reviews, validating it through a case study of five-star hotel [65]. Additionally, some scholars have incorporated time factors to construct a dynamic customer satisfaction model using system dynamics, which has been validated in the automotive industry [66].

Moreover, some studies indicate that external environmental changes influence customer satisfaction dynamics. In a survey of large household appliance purchasing decisions in Romania, COVID-19 is regarded as a potential influencing factor [67]. The impact of COVID-19 on customer satisfaction and its dynamics have been confirmed in studies within the online hospitality and retail industries [32,68]. Existing research indicates that major events, such as the COVID-19 pandemic, have caused temporal shifts in online shopping behavior, leading to dynamic changes in the intrinsic mechanism of online shopping customer satisfaction. Based on the previous research, hypothesis 8 is proposed.

H8: The intrinsic mechanism of online shopping customer satisfaction has differences yearly.

**Fig 2:**
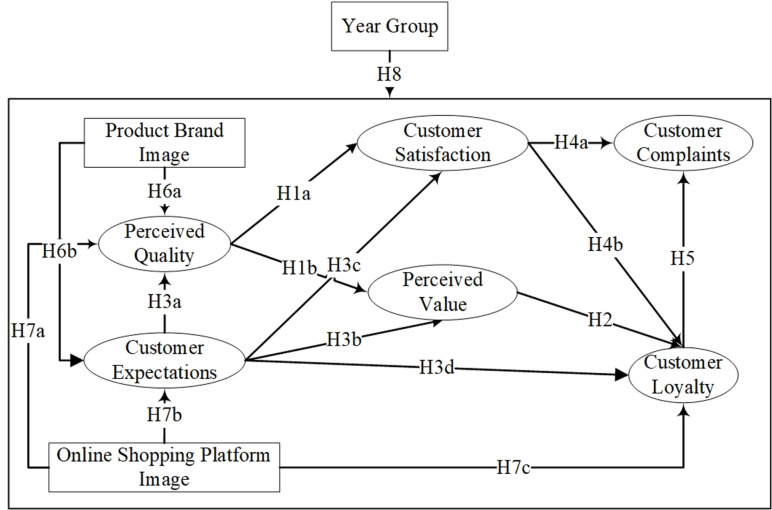
Theoretical model.

Drawing from the insights garnered in the literature review and the research objective, Fig 2 presents the conceptual framework and outlined hypotheses.

## 3. Methodology

To conduct empirical research and test the proposed model (Fig 2), this paper employs positivism philosophy and a deductive theoretical development approach. To operationalize this approach, a structural equation model (SEM) is constructed. The following section will detail the SEM framework and the data sources used.

### 3.1. Research methods

Multi-group SEM is a common and effective method for the analysis of models with moderating effects. It can identify, estimate, and verify various causal models, making it particularly suitable for examining differences in path coefficients across groups and revealing the influence of moderating variables [69,70]. This paper constructs a multi-group SEM based on the theoretical model. It splits a single covariant structure relationship into several parallel covariant structures and then evaluates the equivalence of these covariant structures [71]. Research has shown that sentiment scores from online reviews are highly correlated with customer satisfaction and can effectively represent it [72]. When an observed variable adequately represents a latent variable, a single-indicator construct is appropriate [73]. To better capture latent variables, multiple seed words were used for each observed variable. Previous studies have demonstrated that using a single observed variable to measure latent variables can be both interpretable and valid [74,75]. In marketing, simple perceptions, such as customer preferences for a product or brand, typically do not require multiple items for measurement [76]. Given the characteristics of the data and the representativeness of sentiment scores, using a single-indicator construct is justified. As shown in Table 1, the model includes 8 latent variables and 11 observed variables.

**Table 1:** Latent variables and observed variable.

Latent variables	Symbols	Observed variables
Product brand image	PBI	Customer’s evaluation of the brand image.
Online shopping platform image	OSPI	Customer’s evaluation of the online shopping platform image.
Customer expectations	CE	Customer expectations for product’s quality and price.
Perceived quality	PQ	The customer’s perception of the overall quality of the product (PQ_1_).
		The degree to which the product quality meets customer needs (PQ_2_).
		Customer evaluation of logistics, after-sales service, etc (PQ_3_).
Perceived value	PV	Customer evaluation of the cost-performance of the purchased product.
Customer satisfaction	CS	Overall customer evaluation and rating.
Customer complaints	CC	The degree of customer complaints.
Customer loyalty	CL	Whether the customer repurchases this product (CL_1_).
		Whether the customer recommends others to buy (CL_2_).

Referring to the modeling specification of SEM, we use structural model and measurement model to describe the relationships between variables [77]. The relationships between latent variables are represented by a structural model, see Equation (1). The relationships between latent and observed variables are represented by the measurement model, as shown in Equation (2).


$$\left[\begin{array}{c} \eta_{1}\\ \eta_{2}\\ \eta_{3}\\ \eta_{4}\\ \eta_{5}\\ \eta_{6} \end{array}]=[\begin{array}{cccccc} 0 & 0 & 0 & 0 & 0 & 0\\ \beta_{21} & 0 & 0 & 0 & 0 & 0\\ \beta_{31} & \beta_{32} & 0 & 0 & 0 & 0\\ \beta_{32} & \beta_{42} & 0 & 0 & 0 & 0\\ 0 & 0 & 0 & \beta_{54} & 0 & \beta_{56}\\ \beta_{61} & 0 & \beta_{63} & \beta_{64} & 0 & 0 \end{array}][\begin{array}{c} \eta_{1}\\ \eta_{2}\\ \eta_{3}\\ \eta_{4}\\ \eta_{5}\\ \eta_{6} \end{array}]+[\begin{array}{cc} \alpha_{11} & \alpha_{12}\\ \alpha_{21} & \alpha_{22}\\ 0 & 0\\ 0 & 0\\ 0 & 0\\ 0 & \alpha_{62} \end{array}][\begin{array}{c} \eta_{7}\\ \eta_{8} \end{array}]+[\begin{array}{c} \xi_{1}\\ \xi_{2}\\ \xi_{3}\\ \xi_{4}\\ \xi_{5}\\ \xi_{6} \end{array}\right]$$ (1)

$\eta_{1}-\eta_{8}$ represent CE, PQ, PV, CS, CC, CL, PBI, and OSPI. $\beta_{ij}$ is the structure parameter of the endogenous variable and $\alpha_{ij}\;is\;$the structure parameter of the exogenous variable (i, j takes the value of 1-6), $\xi_{1}-\xi_{8}$ are random errors.


$$\begin{array}{c} \begin{array}{c} \left[\begin{array}{c} {PQ}_{1}\\ {PQ}_{2}\\ {PQ}_{3}\\ {CL}_{1}\\ {CL}_{2} \end{array}\right]=[\begin{array}{ccccc} \lambda_{1} & 0 & 0 & 0 & 0\\ 0 & \lambda_{2} & 0 & 0 & 0\\ 0 & 0 & \lambda_{3} & 0 & 0\\ 0 & 0 & 0 & \lambda_{4} & 0\\ 0 & 0 & 0 & 0 & \lambda_{5} \end{array}]\left[\begin{array}{c} \eta_{2}\\ \eta_{2}\\ \eta_{2}\\ \eta_{6}\\ \eta_{6} \end{array}\right]+\left[\begin{array}{c} \mu_{1}\\ \mu_{2}\\ \mu_{3}\\ \mu_{4}\\ \mu_{5} \end{array}\right]\; \end{array} \end{array}$$ (2)

Where ${PQ}_{1}$ ${PQ}_{2}$ and ${PQ}_{3}$ are observed variables for $\eta_{2}$. ${CL}_{1}$and ${CL}_{2}$ are observation variables for $\eta_{6}$. $\lambda_{1}-\lambda_{5}$ are the latent variable coefficients between observed variables and latent variables. $\mu_{1}-\mu_{5}$ are errors.

The study employs regression equation modeling to estimate the coefficient of direct influence between factors and the satisfaction index of each factor. $Y_{t}$ is the time-series data, which is strongly correlated with time, where t denotes time. Equation (3) is established.


$${\hat{Y}}_{t}=f(t)$$ (3)

From the formula (3), ${\hat{y}}_{t+1}\;$can be obtained.

Equation (4) explains the calculation of the customer satisfaction index (CSI) for each indicator.


$$\begin{array}{c} \text{CSI}=\frac{\sum_{i}^{n}\eta_{i}\times \lambda_{i}}{\sum_{i}^{n}\lambda_{i}} \end{array}$$ (4)

### 3.2. Source of data

This paper uses Python to run and debug the code to collect data, which is derived from publicly available customer reviews on four online shopping platforms: Taobao (https://www.taobao.com/), JD (https://www.jd.com/), Tmall (https://www.tmall.com/), and Suning (https://www.suning.com/). The collection and analysis method complied with the terms and conditions for the source of the data. Online reviews influence customer consumption behavior and reflect customer satisfaction [78,79]. They also include customer satisfaction for different aspects of the products purchased [80]. In terms of market share, JD, Suning, and Tmall together accounted for 95.7% of the B2C market in 2022 [81]. Taobao, a C2C platform, ranks first in market penetration. Major consumer electronics brands, including Apple, Samsung, Huawei, Xiaomi, and OPPO dominate the market. Therefore, we collect online reviews of consumer electronics products from these 5 brands on these 4 e-commerce platforms as our data sources. According to the specific sales situation, we selected 175 representative products. A total number of 267,569 online reviews were collected by August 2022. The collected data include the customer’s review and the date.

To ensure the accurate measurement of variables, we extracted 11 topics using LDA from a large number of reviews, which correspond well to the observed variables (see S1 Appendix). To quantify the observed variables, we employed text sentiment analysis, a method that has recently been widely used to analyze online reviews [82,83]. It refers to the quantification of a text with the subjective sentiment using machine learning or lexicon-based methods [84]. Lexicon-based methods are more suitable for sentence-level sentiment analysis than machine learning [85]. Therefore, we employed lexicon-based text sentiment analysis to quantify online reviews in this study.

**Fig 3:**
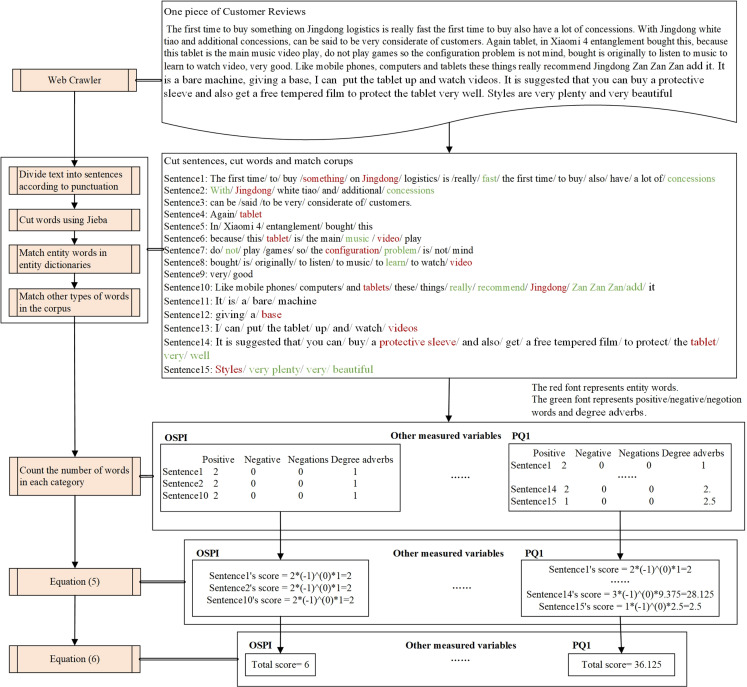
An example of the fine-grained quantization algorithm for text sentiment analysis.


Fig 3 illustrates the quantification algorithm by quantifying a piece of review to the dimension of the OSPI and PQ_1_. The methods used for each phase are described in each sub-process box. A piece of online review collected by the crawler is selected and split into sentences using punctuation and into words using Jieba. We identify the words in the review that match the entity dictionary (see S2 Appendix) for OSPI, PQ_1_ and other measured variables. Subsequently, we detect negations, degree adverbs, as well as positive and negative words in the corresponding sentences using the corpus. The number of each type of word is counted, and each sentence’s score is obtained using equation (5). The review’s score in that dimension is obtained using equation (6). The process of data cleansing and validation is described in S3 Appendix.

$$\begin{array}{c} Each\;sentence^{\prime }s\;score=(\text{P}-\text{N})\times (-1{)}^{n}\times W \end{array}$$ (5)

$$The\;revirew^{\prime }s\;score=\sum Each\;sentence^{\prime }s\;score$$ (6)

P  is the number of positive words. N is the number of negative words. n is the number of negations. W is the weight assigned according to the degree adverbs. The degree equals 1 when the number of degree adverbs is 0.

## 4. Results

**Fig 4:**
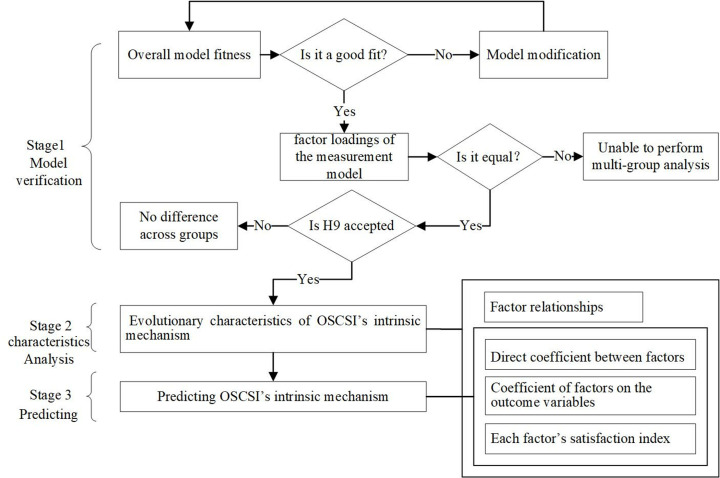
Analysis model of dynamic OSCSI.

The collected data were used to verify and analyze the proposed model, following the procedure outlined in Fig 4. It mainly consists of 3 stages. (1) Model verification: The fitness of the overall model is tested as the foundation for SEM analysis. The equality of factor loadings across years in the measurement model is assessed, which is essential for analyzing the moderating effect. The final test determines whether the year has a moderating effect, serving as a prerequisite for analyzing evolutionary characteristics. (2) Characteristics analysis: The analysis progresses from shallow to deep across four levels: the relationships between factors, the degree of influence among factors, the influence coefficient of each factor on the outcome variables and the satisfaction index of each factor. (3) Prediction: This focuses on the last three aspects of the characteristics analysis. According to the analytical model proposed in Fig 4, each stage is described in detail in the following sub-sections.

### 4.1. Verification of OSCSI

The model was built using Amos 21.0. The testing procedure for the hypothesis, including the bootstrap test added to assess the robustness of the model, is described in S4 Appendix. Table 2 shows that all hypotheses are accepted.

**Table 2:** The result of the hypothesis test.

Hypothesis	Description	Outcome
H1a	Perceived quality directly affects customer satisfaction.	Accept
H1b	Perceived quality directly affects perceived value.	Accept
H2	Perceived value directly affects customer loyalty.	Accept
H3a	Customer expectations directly affect perceived quality.	Accept
H3b	Customer expectations directly affect perceived value.	Accept
H3c	Customer expectations directly affect customer satisfaction.	Accept
H3d	Customer expectations directly affect customer loyalty.	Accept
H4a	Customer satisfaction directly affects customer complaints.	Accept
H4b	Customer satisfaction directly affects customer loyalty.	Accept
H5	Customer loyalty directly affects customer complaints.	
H6a	Product brand image directly affects perceived quality.	Accept
H6b	Product brand image directly affects customer expectations.	Accept
H7a	Online shopping platform image directly affects perceived quality.	Accept
H7b	Online shopping platform image directly affects customer expectations.	Accept
H7c	Online shopping platform image directly affects customer loyalty.	Accept
H8	The intrinsic mechanism of online shopping customer satisfaction has differences yearly.	Accept

The findings indicate that the OSCSI model varies significantly across different years. Therefore, we proceed to analyze the intrinsic mechanism’s evolution characteristics regarding online shopping customer satisfaction.

### 4.2. Evolutionary characteristics of OSCSI’s intrinsic mechanism

#### 4.2.1. Evolutionary characteristics of factor relationships.

The structure of the measurement model and structural model remains stable over time, as shown in Table 3. The path from CS to CL in 2017 is not statistically significant. We attribute this to two reasons. (1) The sample size in 2017 is smaller than that of other years. (2) 2017 witnessed significant shifts in e-commerce platform services, technologies, and products [86]. Changes in the external environment affect the significance of the relationship between CS and CL. In addition, the coefficient of CS on CL has remained relatively low from 2017 to 2022. This result is not only related to the method of data collection, but also reflects a shift in the main drivers of CL. Specifically, while CS remains a significant factor, CL in the online shopping environment is influenced by additional factors, such as CE and OSPI.

**Table 3:** Path coefficients and significance from 2017 to 2022.

Path	2017	2018	2019	2020	2021	2022
CE < --PBI	0.343^***^	0.188^***^	0.271^***^	0.302^***^	0.328^***^	0.339^***^
CE < --OSPI	0.249^***^	0.303^***^	0.623^***^	0.643^***^	0.611^***^	0.594^***^
PQ < --OSPI	0.391^***^	0.287^***^	0.372^***^	0.43^***^	0.431^***^	0.484^***^
PQ < --PBI	0.275^***^	0.292^***^	0.329^***^	0.349^***^	0.331^***^	0.33^***^
PQ < --CE	0.236^***^	0.313^***^	0.237^***^	0.211^***^	0.22^***^	0.149^***^
PV < --PQ	0.304^***^	0.361^***^	0.345^***^	0.286^***^	0.307^***^	0.345^***^
PV < --CE	0.361^***^	0.287^***^	0.452^***^	0.664^***^	0.644^***^	0.6^***^
CL < --CS	0.024^NS^	0.04^***^	0.059^***^	0.019^***^	0.03^***^	0.04^***^
CL < --PV	0.087^***^	0.12^***^	0.147^***^	0.083^***^	0.137^***^	0.167^***^
CL < --CE	0.919^***^	0.854^***^	0.686^***^	0.783^***^	0.712^***^	0.652^***^
CL < --OSPI	0.044^**^	0.134^***^	0.193^***^	0.133^***^	0.152^***^	0.17^***^
CS < --PQ	0.393^***^	0.491^***^	0.667^***^	0.694^***^	0.718^***^	0.689^***^
CS < --CE	0.126^***^	0.054^***^	0.082^***^	0.219^***^	0.098^**^	0.18^***^
CC < --CS	0.115^***^	0.109^***^	0.138^***^	0.198^***^	0.183^***^	0.236^***^
CC < --CL	0.244^***^	0.356^***^	0.586^***^	0.739^***^	0.701^***^	0.643^***^
PQ1 < --PQ	0.36^-^	0.419^-^	0.547^-^	0.591^-^	0.545^-^	0.583^-^
PQ2 < --PQ	0.531^***^	0.451^***^	0.693^***^	0.833^***^	0.808^***^	0.806^***^
PQ3 < --PQ	0.547^***^	0.528^***^	0.726^***^	0.871^***^	0.839^***^	0.835^***^
CL2 < --CL	0.857^-^	0.819^-^	0.911^-^	0.982^-^	0.973^-^	0.973^-^
CL1 < --CL	0.609^***^	0.605^***^	0.844^***^	0.978^***^	0.937^***^	0.948^***^

** Significantly at 0.01 level.

*** Significantly at 0.001 level.

- Path coefficient is constant.

^NS^Path coefficient is not significant.

#### 4.2.2. Evolutionary characteristics of the direct coefficient between factors.

Section 4.2.1 indicates that the relationships between the factors are generally stable. Accordingly, this study proceeds to examine the evolutionary characteristics of the degree of direct influence between these factors.

**Fig 5:**
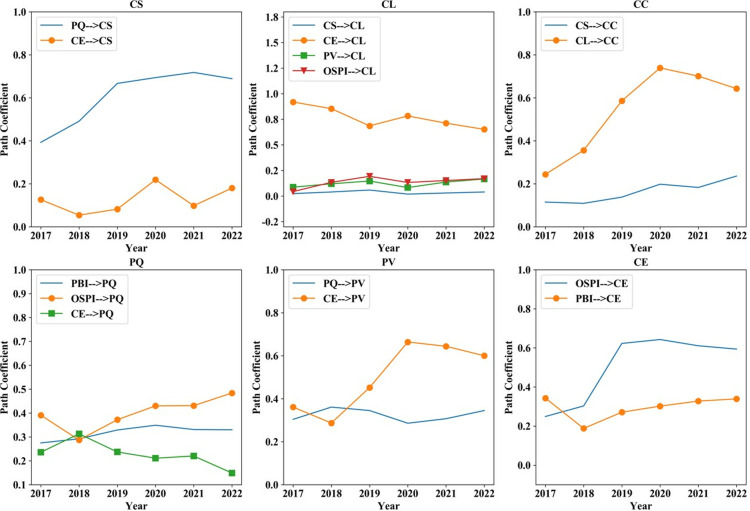
Direct influence coefficient between factors 2017–2022.

Over time, the degree of direct influence between factors exhibits a distinct fluctuation pattern. Some show an increase, while others display “V” or “M” shaped curves, as depicted in Fig 5. Notably, PQ is the primary factor driving CS, while CE predominantly shapes CL, which ultimately drives CC. The overall effect of PQ on CS exhibits an upward trend over time, while the effects of CE on CL and CL on CC both demonstrate a slight decrease following 2020. This indicates that improving the quality of online shopping goods is critical for enhancing CS. Customers exhibiting elevated expectations are predisposed to exhibit greater loyalty. Loyal customers are also more inclined to provide constructive feedback in the form of complaints, thereby assisting the company in improving its products and services.

Following 2018, OSPI emerged as the primary determinant influencing PQ and CE, which subsequently serve as critical factors affecting PV. This indicates that varying OSPI driven by differences in seller rules, logistics services, and product positioning are increasingly significant in shaping customer perceptions. As a result, platforms such as Taobao, JD, and Suning should prioritize the maintenance of their images. Sellers can raise CE through targeted advertising and optimized product homepages, thereby increasing PV.

The COVID-19 pandemic has notably impacted the direct coefficient between factors. The majority of these impacts have been negative shocks, resulting in a reduction of the direct impact coefficients. Specifically, the impact of CE on CL and PV exhibits a declining trend, with 2020 serving as the inflection point. It demonstrates that the epidemic diminished the influence of increased CE on CL and PV, with a similar effect observed for the relationship between CL and CC.

#### 4.2.3. Evolutionary characteristics of the coefficient of factors on the outcome variables.

After studying the evolutionary characteristics of the degree of direct influence between factors, the indirect and total influence coefficients of the independent variables on the dependent variable need to be further explored. S5 Appendix clarifies the direct, indirect, and total impact coefficients of each variable on CS, CL, and CC from 2017 to 2022.

**Fig 6:**
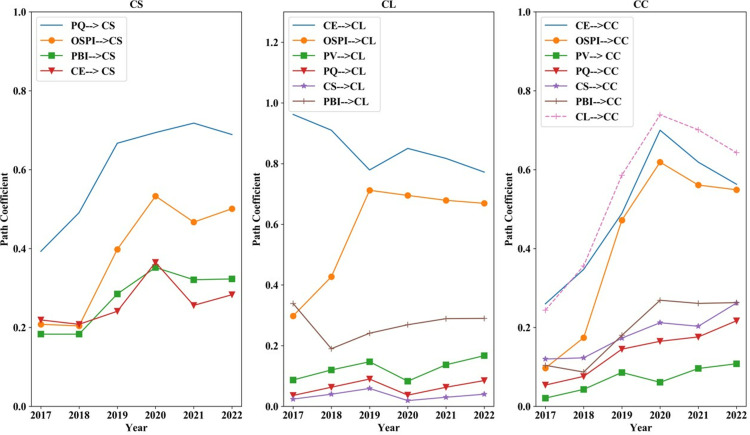
Influence coefficient of factors on outcome variables 2017–2022.

Fig 6 illustrates that PQ has consistently been the most significant factor affecting CS, with its influence gradually increasing over time. The impact of OSPI, PBI and CE on CS shifted from an upward to a downward trend in 2020, with a slight rebound observed in 2022. CE and OSPI have consistently been the two primary factors influencing CL. The impact of OSPI, PV, PQ and CS on CL reached an inflection point in 2019, followed by a marked decline in 2020. It’s worth noticing that CL, CE and OSPI are the three core factors influencing CC, all exerting a positive effect. Enhancing CL, CE, and OSPI can increase the frequency of customer suggestions for product and service improvements in reviews. The positive effects of all three on CC exhibit a decreasing trend after peaking in 2020. This indicates that during the strict epidemic control period, customers became more demanding regarding product and service quality. Improving CL, CE, and OSPI will effectively enhance the frequency of customers articulating their expectations and suggestions for products in reviews.

#### 4.2.4. Evolutionary characteristics of each factor’s satisfaction index.

The study further analyzes each factor’s satisfaction index after exploring the evolution characteristics of the intrinsic structure of OSCSI. It can intuitively reflect whether customers are satisfied or not.

**Fig 7:**
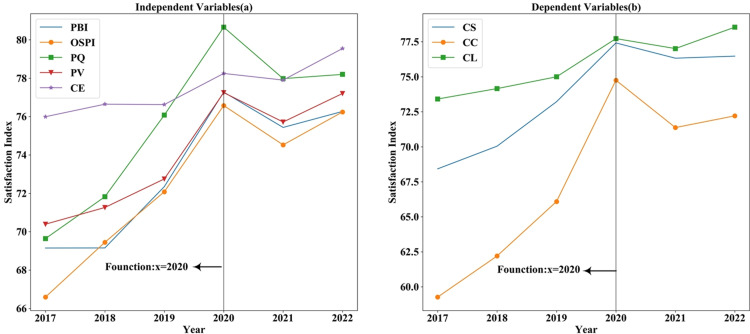
Satisfaction index of each variable 2017–2022.

There is still more room to improve customer satisfaction in online shopping for consumer electronics. The indices of CL, CS, and CC for consumer electronics acquired via online shopping platforms have exhibited an upward trend over time (see Fig 7). In addition, it indicates a gradual increase in the CE of online electronic products, reflecting a growing willingness among customers to articulate their expectations through reviews. Furthermore, PQ, CE, PV, OSPI and PBI exhibited an upward trend until 2020, indicating a growing customer acceptance of online shopping over time. Following the epidemic, a sharp decline occurred in 2021, followed by a slight recovery.

### 4.3. Predicting OSCSI’s intrinsic mechanism

#### 4.3.1. Scenario setting.

The evolutionary characteristics of the intrinsic mechanism of OSCSI reveal a dynamic pattern in the changing direct influence coefficients between factors over time. Based on this observation, we assume that the structure of OSCSI remains stable. Subsequently, two scenarios are established to predict the intrinsic mechanism of OSCSI using regression equations.

The first scenario involves simulating the impact of the pandemic without any societal or enterprise response measures. In this scenario, historical data from 2017 to 2020 is utilized to predict data from 2021 to 2022. The second is the actual scenario, using data from 2017 to 2022 to forecast data from 2023 to 2025.

Certain constraints are implemented to ensure the rationality and accuracy of the predictions. The influence coefficients between factors are restricted to the range of (-1,1), while the factors’ satisfaction index is limited to (0,100). Additionally, the mean of the overall goodness of fit for the prediction model is set to be above 0.5.

#### 4.3.2. Under pandemic impact scenarios.

**Fig 8:**
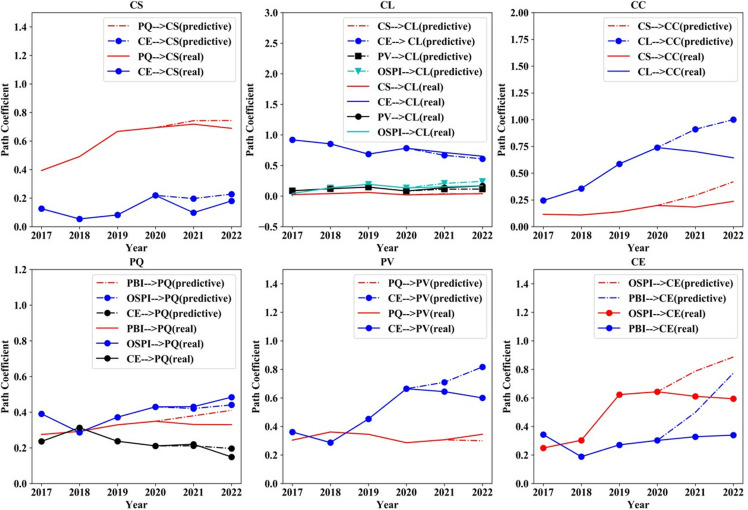
Direct influence coefficient between factors under pandemic impact scenarios 2017–2022.

*4.3.2.1. The direct impact coefficient between factors:* China’s effective pandemic control measures have played a crucial role in maintaining market stability and mitigating the adverse effects on the consumer electronics industry. Fig 8 illustrates that the impact of CS and CL on CC would be significantly enhanced, while the influence of PBI and OSPI on CE would also be increased without COVID-19 interventions. However, if online shopping platforms or brands fail to meet these expectations, customers may become more disappointed, negatively impacting CS and CL, thereby hindering industry development. In terms of the relationship between CE and CL, the epidemic has resulted in a declining coefficient of influence. This suggests that increasing CE at the same cost yields a reduced improvement in CL.

**Fig 9:**
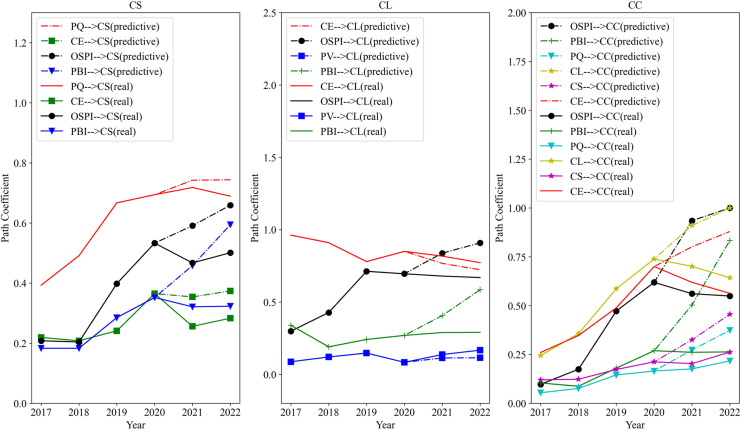
Influence coefficient of factors on outcome variables under pandemic impact scenarios 2017–2022.

*4.3.2.2. The influence coefficient of each factor on the outcome variables:* Fig 9 illustrates that due to the pandemic, the impact of PBI and OSPI on CS, CL and CC is expected to notably escalate in magnitude. This heightened influence is anticipated to reinforce customers’ dependence on brands and platforms. Nevertheless, in the case of an adverse incident involving the brand or platform, the resulting economic repercussions are anticipated to be more pronounced. It is noteworthy that epidemic shocks further diminish the impact of PV and CE on CL. This not only renders marketing riskier and costlier for organizations but also diminishes its efficacy.

**Fig 10:**
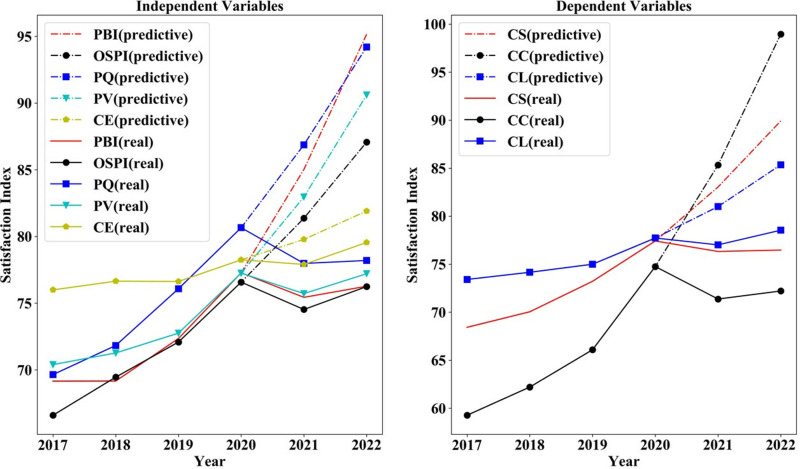
Factors’ satisfaction index under pandemic impact scenarios 2017–2022.

*4.3.2.3. Satisfaction index of each factor:* Fig 10 demonstrates that in the context of the epidemic impact, there is a notable upward trend in the scores for all factors. This may arise from customers’ increased reliance on brands and platforms during exceptional circumstances. Furthermore, while the scores for CS and CL have both risen, the score for CC has exhibited a greater increase, surpassing both CS and CL. This implies that companies should not solely prioritize the short-term enhancements in CS and CL but should also be cautious of the potential risks associated with the rise in CC.

#### 4.3.3. In actual scenarios.

**Fig 11:**
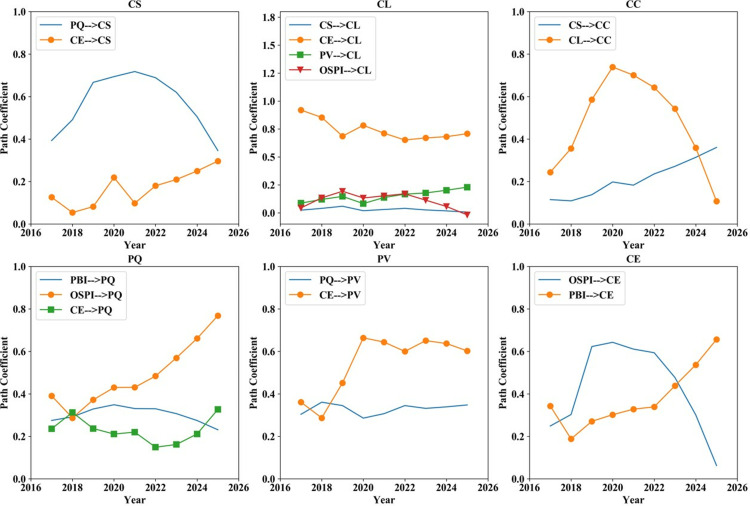
Direct influence coefficient between factors in actual scenarios 2017–2025.

*4.3.3.1. The direct impact coefficient between factors:* Fig 11(CS) and (PQ) suggest that OSPI is the primary factor directly influencing PQ, which further influences CS. Fig 11(CE) shows that the impact of PBI on CE exceeds the impact of OSPI on CE over time. Moreover, Fig 11(CL) illustrates that PV and OSPI are the main factors affecting CE. From Fig. 11(PV) and Fig 11(CL), it is evident that managing CE is crucial as its impact on CL and PV would be significant. Additionally, based on Fig 11(CC), it could be inferred that CS would emerge as the primary determinant of CC in the future, with the influence of CL diminishing.

The analysis suggests that future marketing strategies should focus on three key areas: enhancing the OSPI, improving PQ, and effectively managing PBI. Additionally, optimizing the management of CE and promptly addressing CC can significantly boost overall CS.

**Fig 12:**
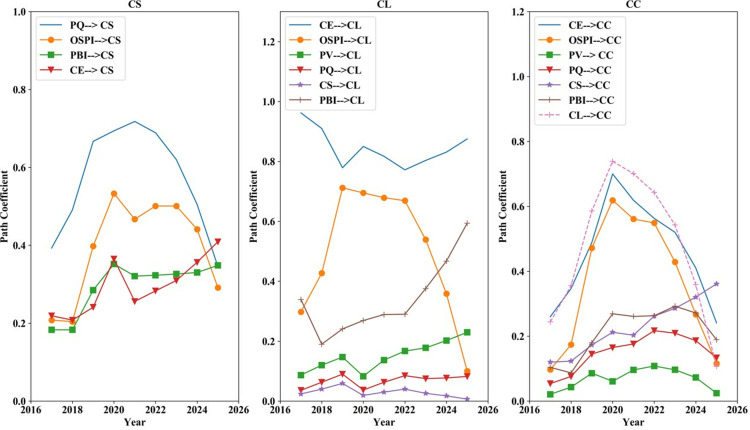
Influence coefficient of factors on outcome variables in actual. Scenarios 2017–2025.

*4.3.3.2. The influence coefficient of each factor on the outcome variables:* According to Fig 12, CE, PQ, and PBI are projected to exert a substantial impact on CS in the future. Furthermore, CE, PBI, and PV have significant impacts on CL. Enhancing both CE and PBI would elevate CC, albeit with a reduced increase compared to previous levels. Therefore, promoting the OSPI and CE should be considered as an additional factor. Improving PQ and the cost-performance ratio are key factors to enhance CS, retain customers, and reduce complaints.

**Fig 13:**
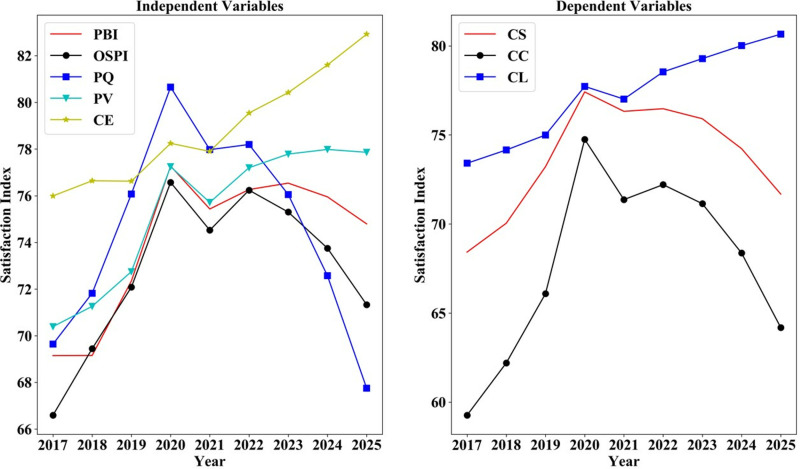
Satisfaction index of each factor in actual scenarios 2017–2025.

*4.3.3.3. Satisfaction index of each factor:* Fig 13 indicates that the scores of all factors, except CE, are projected to decrease, suggesting heightened competition in the consumer electronics sector in the future. Companies will be required to allocate more resources to fulfill the elevated CE. Moreover, the CL score is expected to experience a slight increase in the future, suggesting a growing recognition of the online shopping channel among customers. Both CS and CC are projected to decrease. The decline in CS is intricately tied to the rise in CE, indicating the increasing challenge for companies to meet elevated CE.

## 5. Discussion and implications

A dynamic OSCSI model is developed, integrating CSI and online reviews. The data quantified using fine-grained text sentiment analysis methods can be used as data sources, which is consistent with previous studies [87]. This study confirms the presence of time heterogeneity in the intrinsic mechanism of online customer satisfaction. It enlightens us to analyze the evolutionary characteristics of the intrinsic mechanism of OSCSI from the aspects of the relationship between factors, influence coefficient, and satisfaction index. Notably, we find that the intrinsic mechanism of OSCSI is significantly different before and after the pandemic. It is consistent with previous studies [88]. To further validate these differences and capture the future trend of OSCSI’s intrinsic mechanism, we employe regression equations to predict OSCSI under both pandemic shock and actual scenarios.

This study enriches the literature on marketing management. Firstly, the research adds to the literature of dynamic research on the intrinsic mechanism of customer satisfaction. Previous studies on dynamic customer satisfaction mostly collected data through questionnaires or interviews and primarily analyzed the evolution characteristics of customer satisfaction at the single level of satisfaction index [89]. We further analyze and predict the intrinsic mechanism of OSCSI.

In addition, this study provides a new algorithm for fine-grained quantification of text data. Previous studies on customer satisfaction using online reviews have mostly used word frequency statistics to quantify a single dimension [90,91]. This paper takes a step further. By leveraging web crawlers to collect dynamic data, we address the limitations of static and delayed data obtained through traditional questionnaire methods. To process textual data into structured data, we employ a constructed entity dictionary and utilize regular matching to quantify online reviews across multiple dimensions. Moreover, the test results of the reliability and validity of structured data are good, indicating that it is feasible to obtain data by this method.

### 5.1. Theoretical implications

Firstly, the paper constructed a theoretical model of the intrinsic mechanism of OSCSI and tested the model using quantified text review data. The results reveal that the intrinsic mechanism of OSCSI exhibits greater complexity compared to ACSI.

Secondly, this paper delves into the evolutionary characteristics of the intrinsic mechanism of OSCSI from a temporal perspective, thus extending the existing literature on the dynamics of customer satisfaction. Previous studies primarily focused on establishing dynamic customer satisfaction measurement systems and monitoring them through online reviews [65]. However, this paper goes a step further by integrating textual data with a management model, providing a comprehensive analysis of the subject.

Thirdly, this study presents noteworthy findings in comparison to previous research. Specifically, it recognizes the significant and positive influence of CL on CC. Additionally, the study emphasizes the importance of PBI and OSPI as indicators of product quality in the future.

Lastly, this research confirms the short-term negative impact of Covid-19 on the e-commerce market in line with earlier studies [92]. However, it also reveals that China’s pandemic prevention policies have yielded more favorable outcomes. Despite the challenges posed by Covid-19, e-commerce companies have managed to reverse unfavorable circumstances, and the satisfaction index of all factors will be improved in the post-pandemic era.

### 5.2. Management implications

Firstly, enterprises can enhance CS by concentrating on three key aspects: ensuring PQ, improving PBI, and enhancing the OSPI. It is crucial for enterprises to prioritize CL and effectively address CC, especially loyal customers’ complaints. In today’s discerning market, customers have higher expectations and face more choices, making it imperative for businesses to avoid even minor errors that could result in the loss of loyal customers and an increase in customer complaints.

Secondly, this study offers valuable insights for e-commerce enterprises aiming to adopt data-driven decision-making. The dynamic nature of online shopping customer satisfaction poses risks to marketing decisions within such companies. Fortunately, a vast of online reviews have accumulated on the Internet, serving as a valuable data source for managerial decision-making. To mitigate these risks and make informed marketing decisions, it is imperative for enterprises to leverage big data technology in customer relationship management. By utilizing the existing dynamic data effectively, enterprises can embrace a data-driven decision-making approach and bring about transformative changes in their management practices.

Thirdly, it is essential for e-commerce companies to avoid potential pitfalls when formulating future marketing strategies. Specifically, the conventional approach of solely focusing on enhancing PBI, and OSPI may not effectively improve CL in the long run. While these factors can serve as supporting elements, they should not overshadow the significance of PQ. Neglecting these crucial aspects can undermine efforts to improve CS in the future, rendering it a weak point that needs to be addressed.

Lastly, the COVID-19 pandemic has undeniably presented challenges to the consumer electronics industry in the realm of online shopping. However, it also brings forth both challenges and opportunities. E-commerce companies are advised to collaborate with the relevant policies implemented by the Chinese government, swiftly adapt to the evolving external environment, and make necessary adjustments to their marketing strategies. A key focus should be on accentuating the strengths of the brand image associated with their products and improving PQ. By doing so, the adverse effects of the pandemic on the consumer electronics industry can be mitigated effectively.

## 6. Conclusions, limitations and future research

This paper explores the evolution characteristics of the intrinsic mechanism of OSCSI, focusing on consumer electronic products as a case study. The intrinsic mechanism of OSCSI is predicted under pandemic shock scenarios and actual scenarios. It is found that the relationships between the factors of the intrinsic mechanism of OSCSI are stable. The degree of direct influence among factors shows temporal correlation, with notable inflection points or peaks observed in 2020. Notably, the impact coefficient of PQ on CS shows a steady annual increase, with a slight decline in 2022. The impact of CE and OSPI on CC and CL fluctuates significantly with time. The study also indicates that both PBI and OSPI directly enhance PQ, while emphasizing the amplifying effect of CL on CC. Furthermore, the research identifies the negative impact of COVID-19 on online shopping for consumer electronics, which has been partially alleviated by China’s pandemic prevention policies. By conducting this analysis, we contribute to a deeper understanding of OSCSI and its dynamics, shedding light on the impacts of external factors and providing insights for future research and practical applications.

There are some limitations in this study. First, this study is constrained by the platform when collecting data, resulting in an uneven number of reviews collected across platforms and brands. Second, this study does not incorporate multidimensional measurement variables to analyze the underlying causes of changes in the intrinsic mechanism of OSCSI over time.

We provide two research directions for future scholars. Firstly, future research can collect more sufficient data to compare the temporal and spatial characteristics of OSCSI from the perspective of platforms and brands to discover the differences and insights. Secondly, we can further investigate the underlying reasons for changes in the intrinsic mechanism, so as to provide specific countermeasures for e-commerce companies to improve CS and retain customers.

## Supporting information

S1 Appendix Latent Dirichlet Allocation (LDA) topic model. (DOCX)

S2 Appendix Entity dictionary.csv. (DOCX)

S3 Appendix Data cleaning and validation procedures. (DOCX)

S4 Appendix Hypothesis testing and bootstrap resampling procedure. (DOCX)

S5 Appendix The direct, indirect, and total impact coefficients. (CSV)
