# Supplementary material for: Probing the intrinsic mechanism and evolution characteristics of online shopping customer satisfaction via text mining of online reviews
Source: PLoS One. 2025 May 7;20(5):e0321202. doi: 10.1371/journal.pone.0321202 (PMC12058191; doi:10.1371/journal.pone.0321202)
Supplement: S1 Appendix — (DOCX) [file pone.0321202.s001.docx]

# Supplementary Materials

## Appendix A： Latent Dirichlet Allocation (LDA) Topic Model

To enhance the modeling quality of the LDA topic model, we employed a word cloud visualization tool. A total of 157 words, each with a frequency exceeding 3000, were selected to generate the corresponding word cloud (see Fig. 1), where the word size is proportional to its frequency. These high-frequency terms capture various dimensions of customer satisfaction. For instance, words such as “running,” “battery life,” and “function” reflect the perceived quality, while “price” and “price reduction” represent perceived value.


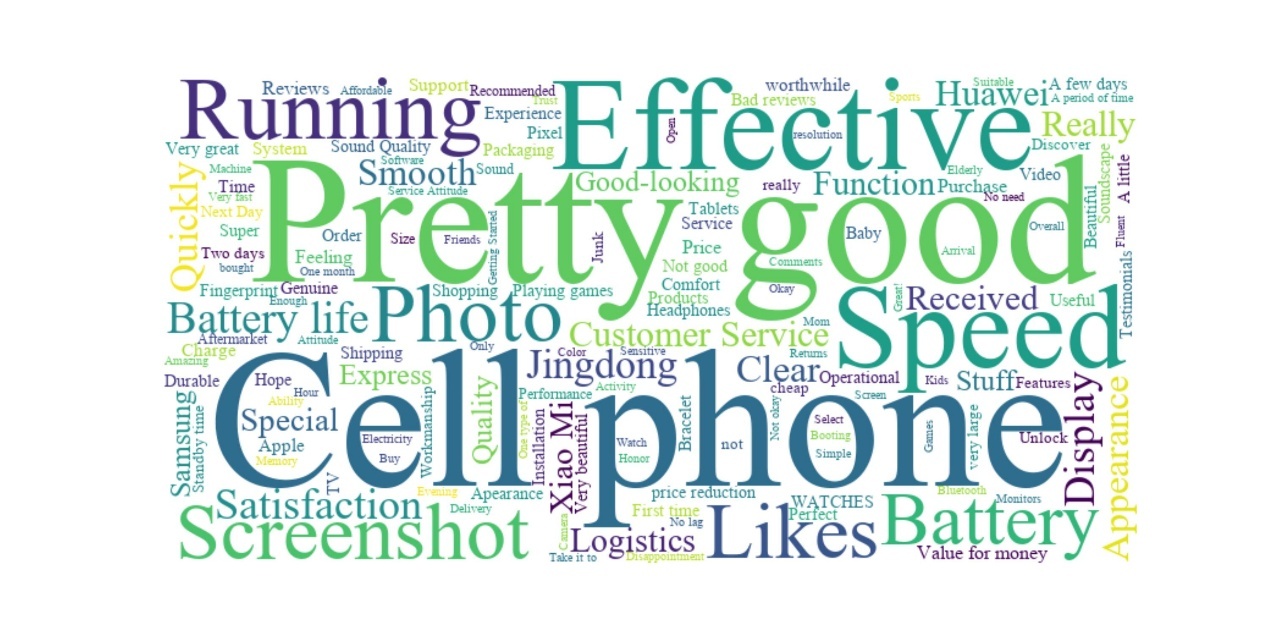


Fig. 1. Word Cloud for the high-frequency comments in the English version

To explore the relationships between words and topics, we employ large-scale language modeling to reveal hidden connections. Specifically, we use LDA topic models from the Gensim library to mine potential patterns and associations in text data. This approach identifies the core topics expressed by consumers through their reviews and extracts high-frequency words that are closely related to these topics, thus reflecting the core influences on customer satisfaction.

Before performing LDA topic modeling, it is essential to determine the appropriate number of topics. In this study, the optimal number of topics is determined using perplexity (see Equation 1). As shown in Fig. 2, increasing the number of topics significantly reduces the perplexity, indicating improved model fit [1]. The perplexity reaches its minimum when the number of topics is 11, suggesting the best topic clustering. Additionally, with 11 topics, the model's coherence is approximately 0.6, indicating good topic quality and semantic consistency. Considering both perplexity and coherence, we selected 11 topics to balance prediction accuracy and semantic coherence, aligning with the observed variables in the online shopping customer satisfaction index model.

$$\begin{aligned} \mathrm{Perplexity}=\exp\left( -\frac{\sum_{d=1}^{\left| D \right|} logP\left( w_{d} \right)}{\sum_{d=1}^{\left| D \right|} N_{d}} \right)\#\left( 1 \right) \end{aligned}$$

Where $D$ represents the set of documents, $w_{d}$​ denotes the words in document $d$, $P(w_{d})$ is the probability of words according to the LDA model, and $N_{d}$​ is the number of words in document $d$.


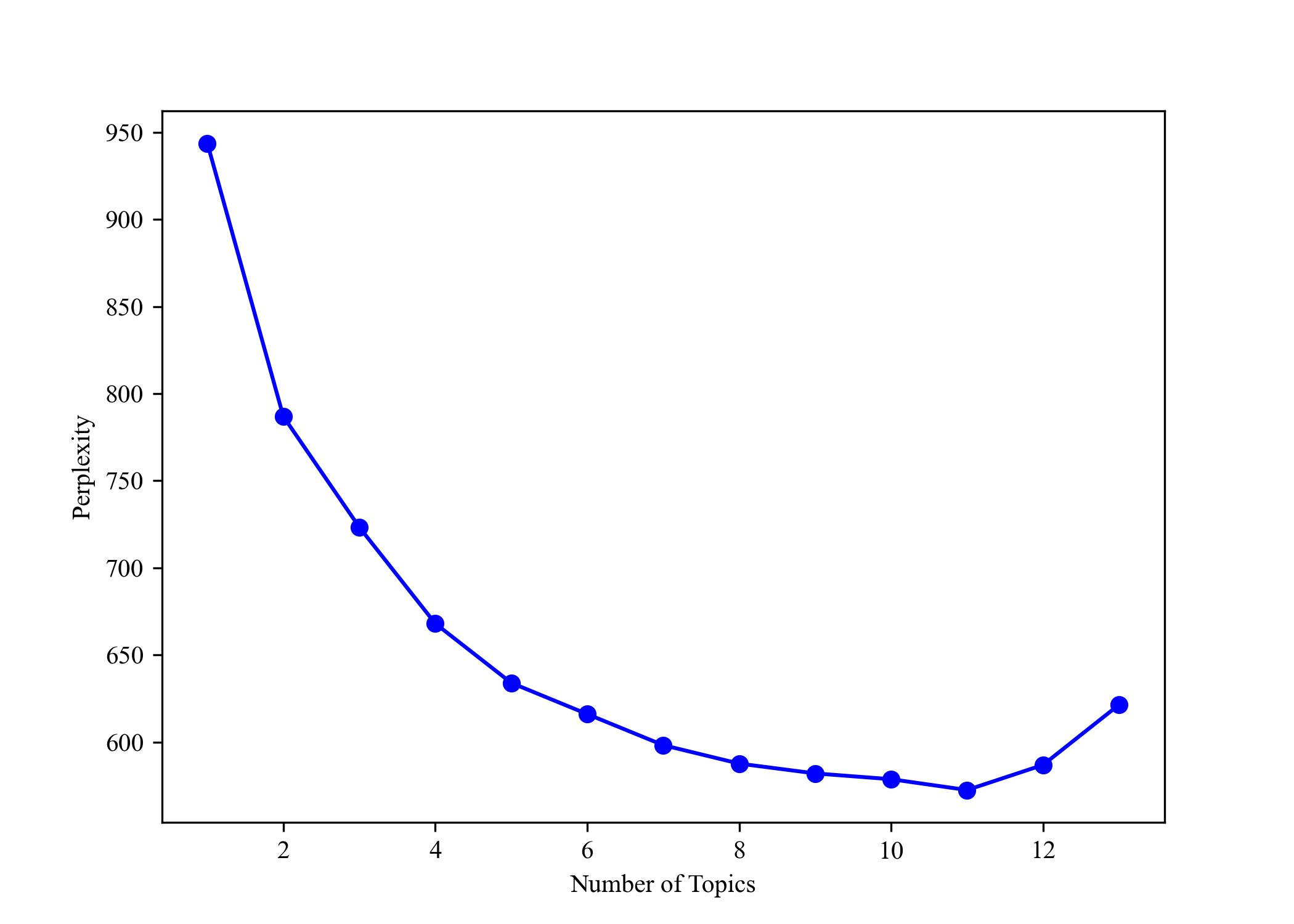


Fig. 2. LDA model perplexity for different number of topics

Based on high-frequency words and core related words with higher weights in each topic, we manually identified 11 topics and their corresponding seed words for sentiment analysis, as shown in Fig. 3. As depicted, some high-frequency words are specific to certain topics, while others are shared across multiple topics, indicating potential correlations between them. This vocabulary sharing reflects the multidimensional nature of customer satisfaction and suggests that customers may consider multiple factors in their evaluation. Therefore, sentiment analysis using these seed words helps capture emotional tendencies in customer online reviews, providing strong data support for subsequent model validation.


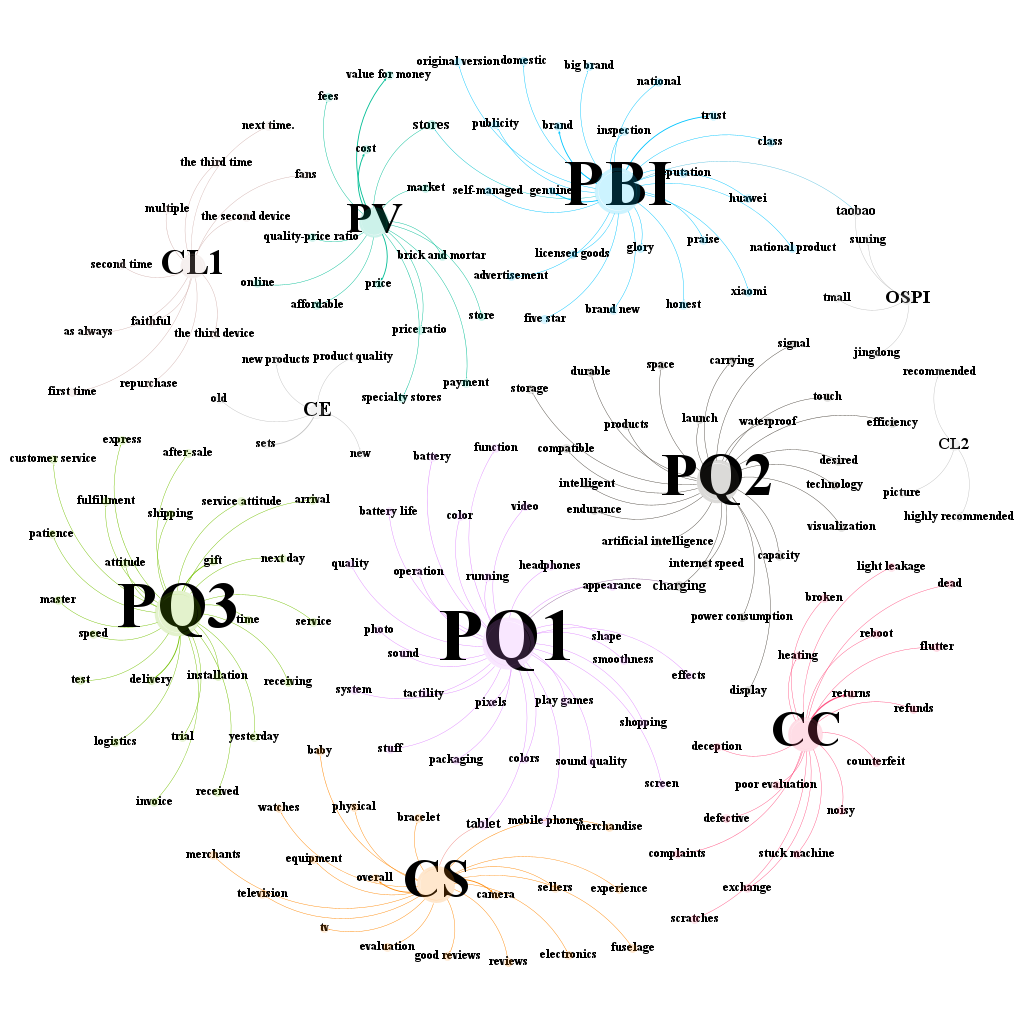


Fig. 3. Topic-High frequency words relationship graph of LDA in English Version

## References

1. Liu X, Zhou Y, Wang Z, Kumar A, Biswas B. Disease topic modeling of users’ inquiry texts: a text mining-based PQDR-LDA model for analyzing the online medical records. IEEE Trans Eng Manag. 2024;71: 6319–6337. doi:10.1109/TEM.2023.3307550
