# Supplementary material for: Probing the intrinsic mechanism and evolution characteristics of online shopping customer satisfaction via text mining of online reviews
Source: PLoS One. 2025 May 7;20(5):e0321202. doi: 10.1371/journal.pone.0321202 (PMC12058191; doi:10.1371/journal.pone.0321202)
Supplement: S2 Appendix — (DOCX) [file pone.0321202.s002.docx]

# Supplementary Materials

## Appendix B：Data Cleaning and validation procedures

**1. Data Cleaning**

A total of 267,569 online reviews were collected, and the data was cleaned based on the principles of non-empty and non-duplicate after excluding the default positive reviews. To ensure data integrity and the effectiveness of the analysis, this study carefully screened and processed the review data. First, each review was quantified across 11 dimensions. To guarantee sufficient information across multiple dimensions, we excluded reviews with fewer than 4 dimensions, leaving a dataset of 36,705 entries. Next, to minimize the potential impact of sample size discrepancies across different years, we excluded data from 2014 to 2016 where the sample size was fewer than 1,000. This resulted in a total of 36,298 reviews for the overall structural equation model analysis. For the multi-group analysis, additional adjustments were made to account for the large sample size in 2019. Specifically, reviews with fewer than 6 dimensions in the 2019 dataset were excluded to ensure that the final sample met the requirements for the analysis. Detailed sample sizes are provided in Tab. 1.

Tab. 1. Annual data volume 2017 - 2022

| Year | 2017 | 2018 | 2019 | 2020 | 2021 | 2022 | Total |
| --- | --- | --- | --- | --- | --- | --- | --- |
| Quantity | 1355 | 7435 | 7603 | 3230 | 2442 | 6236 | 28301 |

After quantifying online reviews, it was found that the data were basically distributed between -6 and 6. According to the 3 sigma principle, outliers beyond the boundary were transferred to -6 or 6. The missing values were supplemented using the concentrated trends of different online shopping platforms [1]. The data is normalized to between 0 and 100 using the range normalization method.

**2. Data Verification**

Cronbach's α was used to assess the reliability of the scale. A higher α value indicates greater reliability and internal consistency of the data. The results show that all Cronbach's α values exceed 0.7, demonstrating good reliability. Validity was evaluated through convergent and discriminant validity analyses. As shown in Tab. 2, the AVE (Average Variance Extracted) values for each scale exceed 0.5, and the CR (Composite Reliability) values are all above 0.7, indicating good convergent validity. Discriminant validity was evaluated by comparing the square root of each latent variable's AVE with the correlation coefficients between the variable and other latent variables. Additionally, the correlation coefficients between any other two variables are less than the square root of their AVE, suggesting that the different variables exhibit good discriminant validity.

Tab. 2. Confirmatory factor analysis and discriminant validity test

|  | CC | CE | CL | CS | OSPI | PBI | PQ | PV | AVE | CR | Cronbach’s α |
| --- | --- | --- | --- | --- | --- | --- | --- | --- | --- | --- | --- |
| CC | 1.00 |  |  |  |  |  |  |  |  |  |  |
| CE | 0.69 | 1.00 |  |  |  |  |  |  |  |  |  |
| CL | 0.72 | 0.93 | 0.96 |  |  |  |  |  | 0.93 | 0.96 | 0.92 |
| CS | 0.59 | 0.63 | 0.65 | 1.00 |  |  |  |  |  |  |  |
| OSPI | 0.69 | 0.78 | 0.80 | 0.61 | 1.00 |  |  |  |  |  |  |
| PBI | 0.63 | 0.70 | 0.73 | 0.60 | 0.68 | 1.00 |  |  |  |  |  |
| PQ | 0.68 | 0.65 | 0.67 | 0.63 | 0.67 | 0.63 | 0.80 |  | 0.64 | 0.84 | 0.73 |
| PV | 0.65 | 0.79 | 0.80 | 0.59 | 0.70 | 0.66 | 0.62 | 1.00 |  |  |  |

Note: The diagonals represent the square root of AVE and the off-diagonals represent the correlation.

## References

1. Xiong, Z. M., Guo, H. Y., Wu, Y. X. Review of missing data processing methods. Computer Engineering and Applications. 2021;57: 27–38. <http://cea.ceaj.org/CN/10.3778/j.issn.1002-8331.2101-0187>.
