# Supplementary material for: Probing the intrinsic mechanism and evolution characteristics of online shopping customer satisfaction via text mining of online reviews
Source: PLoS One. 2025 May 7;20(5):e0321202. doi: 10.1371/journal.pone.0321202 (PMC12058191; doi:10.1371/journal.pone.0321202)
Supplement: S3 Appendix — (DOCX) [file pone.0321202.s003.docx]

# Supplementary Materials

## Appendix C：Hypothesis Testing and Bootstrap Resampling Procedure

**1. Hypothesis testing**

After inputting the data into the model, the root mean square error of approximation (RMSEA) was found to exceed 0.1, indicating inadequate model fit. Informed by the model modification indices and grounded in expectation-disconfirmation theory, we introduced a correlation between the residuals of CE and CC, resulting in an improved model fit.

The goodness-of-fit test results for the structural equation model indicate that the Chi-square value is significant, suggesting a poor fit between the theoretical model and the data. However, Chi-square value is very sensitive to the sample size. The larger the sample size, the less significant the Chi-square value is, resulting in the greater the probability of rejection of the theoretical model. Due to the large sample size of 28,301 used in this study, it was only employed as a reference index for model evaluation. The RMSEA is 0.097, indicating an average fit of the model. The goodness-of-fit index (GFI), normed-fit-index (NFI), relative-fit-index (RFI), incremental-fit-index (IFI), tucker-lewis index (TLI), and comparative fit index (CFI) are 0.946, 0.962, 0.939, 0.962, 0.939, and 0.962, respectively, all of which exceed 0.9. Additionally, parsimonious normed fit index (PNFI) and parsimonious comparative fit index (PCFI) are both 0.595, exceeding the threshold of 0.5. Overall, the model demonstrates a good fit. Furthermore, the factor loadings for each item exceed the recommended value of 0.5, indicating that the selected measurement variables effectively reflect the latent variables. Tab. 3 shows that the path coefficients of both the measurement and structural models are significant, confirming that hypotheses 1 to 9 are accepted.

Tab. 3. The path coefficient and significance of overall model

| Path | Weight and Significance | Path | Weight and Significance |
| --- | --- | --- | --- |
| CE<--PBI | 0.311^***^ | CL<--CE | 0.757^***^ |
| CE<--OSPI | 0.549^***^ | CL<--PV | 0.135^***^ |
| PQ<--PBI | 0.324^***^ | CL<--OSPI | 0.130^***^ |
| PQ<--OSPI | 0.383^***^ | CC<--CS | 0.094^***^ |
| PQ<--CE | 0.240^***^ | CC<--CL | 0.769^***^ |
| PV<--PQ | 0.362^***^ | PQ1<--PQ | 0.513^-^ |
| CS<--PQ | 0.619^***^ | PQ2<--PQ | 0.687^***^ |
| PV<--CE | 0.488^***^ | PQ3<--PQ | 0.708^***^ |
| CS<--CE | 0.126^***^ | CL2<--CL | 0.943^-^ |
| CL<--CS | 0.049^***^ | CL1<--CL | 0.932^***^ |

Note: *** Significantly at 0.001 level. -Path coefficient is constant.

2. Moderating effect testing

To verify the moderating effect of time on OSCSI, a multi-group analysis was conducted. It is typically assumed that the relationships between the latent variables in the structural equation model are consistent across different groups. The measurement weighted model (M1), structural weighted model (M2), structural covariance model (M3), structural error model (M4), and measurement error model (M5) were employed to assess whether time moderates the relationships among the latent variables.

The goodness-of-fit test results for the unconstrained model indicate that the GFI, AGFI, RMESA and CFI are 0.947, 0.907, 0.037 and 0.967, respectively. These values suggest that the model has good fit and that the variables exhibit strong structural validity, making it suitable for multi-group analysis. Based on the goodness-of-fit test results for M1, the GFI, AGFI, RMSEA, and CFI are 0.944, 0.912, 0.036, and 0.965, respectively. These values show that the loadings of measurement factors are consistent across groups, thereby allowing for multi-group difference analysis.

From Tab. 4, it can be observed that the difference values of the different parameters all exceed 1.96. Additionally, based on Tab. 5, assuming equal measurement weights, all p-values are less than 0.05, and the variation values for NFI, IFI, RFI, and TLI exceed 0.01, with some approaching or exceeding 0.05. These results indicate that time has a significant moderating effect, leading to the acceptance of H10.

Tab. 4. critical ratios for differences between parameters (unconstrained)

|  | a2_2 | a3_2 | a4_2 | b5_2 | b9_2 | b8_2 | b1_2 | a7_2 | b2_1 |
| --- | --- | --- | --- | --- | --- | --- | --- | --- | --- |
| a2_2 | 0 |  |  |  |  |  |  |  |  |
| a3_2 | 3.449 | 0 |  |  |  |  |  |  |  |
| a4_2 | -0.901 | -3.362 | 0 |  |  |  |  |  |  |
| b5_2 | -10.849 | -11.012 | -24.639 | 0 |  |  |  |  |  |
| b9_2 | -10.374 | -10.657 | -22.887 | 3.11 | 0 |  |  |  |  |
| b8_2 | -7.658 | -8.51 | -16.83 | 8.59 | 6.48 | 0 |  |  |  |
| b1_2 | -8.118 | -8.89 | -17.304 | 6.472 | 4.623 | -1.257 | 0 |  |  |
| a7_2 | -2.335 | -4.859 | -1.424 | 8.206 | 7.748 | 5.475 | 5.89 | 0 |  |
| b2_1 | -8.724 | -9.409 | -18.534 | 11.812 | 8.351 | -0.358 | 1.003 | -6.138 | 0 |

Tab. 5. Assuming model Measurement weights to be correct

| Model | DF | CMIN | P | NFI Delta-1 | IFI Delta-2 | RFI rho-1 | TLI rho2 |
| --- | --- | --- | --- | --- | --- | --- | --- |
| Structural weights | 55 | 5568.106 | .000 | .021 | .021 | .011 | .011 |
| Structural covariances | 70 | 10364.543 | .000 | .038 | .038 | .026 | .026 |
| Structural residuals | 81 | 12360.618 | .000 | .046 | .046 | .030 | .031 |
| Measurement residuals | 116 | 21639.668 | .000 | .080 | .080 | .053 | .053 |

**2.** Bootstrap Resampling Procedure

To mitigate the potential risks associated with single indicator constructs, this study applied the Bootstrap method to further assess the model's robustness. The results of 5000 bootstrap samples (see Tab. 6) indicate that the 95% confidence intervals of all path coefficients do not include 0, and the p-values are 0, demonstrating that the path coefficients are statistically significant and unaffected by sampling variability. This confirms the robustness of the model and the reliability of the path relationships. Overall, both the measurement and structural models exhibit strong robustness, with the scale design effectively capturing the true characteristics of the latent variables, thus providing a solid foundation for subsequent structural model analysis.

Tab. 6. Path Coefficients with Bootstrap Confidence Intervals

| Path | Lower | Upper | P | Path | Lower | Upper | P |
| --- | --- | --- | --- | --- | --- | --- | --- |
| CE<---PBI | 0.299 | 0.322 | 0 | CL<---CE | 0.743 | 0.779 | 0 |
| CE<---OSPI | 0.536 | 0.561 | 0 | CL<---PV | 0.123 | 0.147 | 0 |
| PQ<---PBI | 0.311 | 0.338 | 0 | CL<---OSPI | 0.113 | 0.138 | 0 |
| PQ<---OSPI | 0.367 | 0.399 | 0 | CC<---CS | 0.082 | 0.108 | 0 |
| PQ<---CE | 0.222 | 0.257 | 0 | CC<---CL | 0.756 | 0.783 | 0 |
| PV<---PQ | 0.344 | 0.381 | 0 | PQ1<---PQ | 0.503 | 0.524 | 0 |
| CS<---PQ | 0.599 | 0.638 | 0 | PQ2<---PQ | 0.679 | 0.695 | 0 |
| PV<---CE | 0.468 | 0.507 | 0 | PQ3<---PQ | 0.701 | 0.715 | 0 |
| CS<---CE | 0.106 | 0.146 | 0 | CL2<---CL | 0.926 | 0.937 | 0 |
| CL<---CS | 0.04 | 0.054 | 0 | CL1<---CL | 0.851 | 0.866 | 0 |
