# Supplementary material for: Probing the intrinsic mechanism and evolution characteristics of online shopping customer satisfaction via text mining of online reviews
Source: PLoS One. 2025 May 7;20(5):e0321202. doi: 10.1371/journal.pone.0321202 (PMC12058191; doi:10.1371/journal.pone.0321202)
Supplement: S4 Appendix — (DOCX) [file pone.0321202.s004.docx]

**Supplementary Materials**

## Appendix D：The direct, indirect, and total impact coefficients

Tab. 7. Influence coefficients of independent variables on customer satisfaction

| Latent variables | Category | 2017 | 2018 | 2019 | 2020 | 2021 | 2022 |
| --- | --- | --- | --- | --- | --- | --- | --- |
| PBI | direct | 0 | 0 | 0 | 0 | 0 | 0 |
|  | indirect | 0.183 | 0.183 | 0.285 | 0.352 | 0.321 | 0.323 |
|  | total | 0.183 | 0.183 | 0.285 | 0.352 | 0.321 | 0.323 |
| OSPI | direct | 0 | 0 | 0 | 0 | 0 | 0 |
|  | indirect | 0.208 | 0.204 | 0.398 | 0.533 | 0.467 | 0.501 |
|  | total | 0.208 | 0.204 | 0.398 | 0.533 | 0.467 | 0.501 |
| CE | direct | 0.126 | 0.054 | 0.082 | 0.219 | 0.098 | 0.18 |
|  | indirect | 0.093 | 0.154 | 0.159 | 0.146 | 0.158 | 0.103 |
|  | total | 0.219 | 0.208 | 0.241 | 0.365 | 0.256 | 0.283 |
| PQ | direct | 0.393 | 0.491 | 0.667 | 0.694 | 0.718 | 0.689 |
|  | indirect | 0 | 0 | 0 | 0 | 0 | 0 |
|  | total | 0.393 | 0.491 | 0.667 | 0.694 | 0.718 | 0.689 |

Tab. 8. Influence coefficients of independent variables on customer loyalty

| Latent variables | Category | 2017 | 2018 | 2019 | 2020 | 2021 | 2022 |
| --- | --- | --- | --- | --- | --- | --- | --- |
| PBI | direct | 0 | 0 | 0 | 0 | 0 | 0 |
|  | indirect | 0.339 | 0.19 | 0.241 | 0.269 | 0.289 | 0.29 |
|  | total | 0.339 | 0.19 | 0.241 | 0.269 | 0.289 | 0.29 |
| OSPI | direct | 0.044 | 0.134 | 0.193 | 0.133 | 0.152 | 0.17 |
|  | indirect | 0.254 | 0.293 | 0.519 | 0.562 | 0.527 | 0.499 |
|  | total | 0.298 | 0.427 | 0.712 | 0.695 | 0.679 | 0.669 |
| CE | direct | 0.919 | 0.854 | 0.686 | 0.783 | 0.712 | 0.652 |
|  | indirect | 0.043 | 0.056 | 0.093 | 0.067 | 0.105 | 0.12 |
|  | total | 0.962 | 0.91 | 0.779 | 0.85 | 0.817 | 0.772 |
| PQ | direct | 0 | 0 | 0 | 0 | 0 | 0 |
|  | indirect | 0.036 | 0.063 | 0.09 | 0.037 | 0.063 | 0.085 |
|  | total | 0.036 | 0.063 | 0.09 | 0.037 | 0.063 | 0.085 |
| CS | direct | 0.024 | 0.04 | 0.059 | 0.019 | 0.03 | 0.04 |
|  | indirect | 0 | 0 | 0 | 0 | 0 | 0 |
|  | total | 0.024 | 0.04 | 0.059 | 0.019 | 0.03 | 0.04 |
| PV | direct | 0.087 | 0.12 | 0.147 | 0.083 | 0.137 | 0.167 |
|  | indirect | 0 | 0 | 0 | 0 | 0 | 0 |
|  | total | 0.087 | 0.12 | 0.147 | 0.083 | 0.137 | 0.167 |

Tab. 9. Influence coefficient of independent variables on customer complaints

| Latent variables | Category | 2017 | 2018 | 2019 | 2020 | 2021 | 2022 |
| --- | --- | --- | --- | --- | --- | --- | --- |
| PBI | direct | 0 | 0 | 0 | 0 | 0 | 0 |
|  | indirect | 0.104 | 0.087 | 0.18 | 0.269 | 0.261 | 0.263 |
|  | total | 0.104 | 0.087 | 0.18 | 0.269 | 0.261 | 0.263 |
| OSPI | direct | 0 | 0 | 0 | 0 | 0 | 0 |
|  | indirect | 0.097 | 0.174 | 0.472 | 0.619 | 0.561 | 0.549 |
|  | total | 0.097 | 0.174 | 0.472 | 0.619 | 0.561 | 0.549 |
| CE | direct | 0 | 0 | 0 | 0 | 0 | 0 |
|  | indirect | 0.26 | 0.347 | 0.489 | 0.7 | 0.619 | 0.563 |
|  | total | 0.26 | 0.347 | 0.489 | 0.7 | 0.619 | 0.563 |
| PQ | direct | 0 | 0 | 0 | 0 | 0 | 0 |
|  | indirect | 0.054 | 0.076 | 0.145 | 0.165 | 0.176 | 0.217 |
|  | total | 0.054 | 0.076 | 0.145 | 0.165 | 0.176 | 0.217 |
| CS | direct | 0.115 | 0.109 | 0.138 | 0.198 | 0.183 | 0.236 |
|  | indirect | 0.005 | 0.014 | 0.035 | 0.014 | 0.02 | 0.026 |
|  | total | 0.12 | 0.123 | 0.173 | 0.212 | 0.203 | 0.262 |
| PV | direct | 0 | 0 | 0 | 0 | 0 | 0 |
|  | indirect | 0.021 | 0.043 | 0.086 | 0.061 | 0.096 | 0.108 |
|  | total | 0.021 | 0.043 | 0.086 | 0.061 | 0.096 | 0.108 |
| CL | direct | 0.244 | 0.356 | 0.586 | 0.739 | 0.701 | 0.643 |
|  | indirect | 0 | 0 | 0 | 0 | 0 | 0 |
|  | total | 0.244 | 0.356 | 0.586 | 0.739 | 0.701 | 0.643 |
